# Drug Repurposing for the Identification of Compounds with Anti-SARS-CoV-2 Capability via Multiple Targets

**DOI:** 10.3390/pharmaceutics14010176

**Published:** 2022-01-12

**Authors:** Pei-Chen Yu, Chen-Hao Huang, Chih-Jung Kuo, Po-Huang Liang, Lily Hui-Ching Wang, Max Yu-Chen Pan, Sui-Yuan Chang, Tai-Ling Chao, Si-Man Ieong, Jun-Tung Fang, Hsuan-Cheng Huang, Hsueh-Fen Juan

**Affiliations:** 1 Department of Life Science and Institute of Molecular and Cellular Biology, National Taiwan University, Taipei 10617, Taiwan; d05b43001@ntu.edu.tw; 2 Graduate Institute of Biomedical Electronics and Bioinformatics, National Taiwan University, Taipei 10617, Taiwan; d08945005@ntu.edu.tw; 3 Department of Veterinary Medicine, National Chung Hsing University, Taichung 40227, Taiwan; ck476@dragon.nchu.edu.tw; 4 Institute of Biological Chemistry, Academia Sinica, Taipei 11529, Taiwan; phliang@gate.sinica.edu.tw; 5 Institute of Biochemical Sciences, National Taiwan University, Taipei 10617, Taiwan; 6 Institute of Molecular and Cellular Biology, National Tsing Hua University, Hsinchu 30004, Taiwan; lilywang@life.nthu.edu.tw (L.H.-C.W.); maxsos1996@hotmail.com (M.Y.-C.P.); 7 Department of Clinical Laboratory Sciences and Medical Biotechnology, National Taiwan University, Taipei 10048, Taiwan; sychang@ntu.edu.tw (S.-Y.C.); d01424001@ntu.edu.tw (T.-L.C.); ccccman86@gmail.com (S.-M.I.); kkjjkk870619@gmail.com (J.-T.F.); 8 Department of Laboratory Medicine, National Taiwan University Hospital, Taipei 10002, Taiwan; 9 Genomics Research Center, Academia Sinica, Taipei 11529, Taiwan; 10 Institute of Biomedical Informatics, National Yang Ming Chaio Tung University, Taipei 11230, Taiwan; 11 Center for Computational and Systems Biology, National Taiwan University, Taipei 10617, Taiwan

**Keywords:** docking simulation, severe acute respiratory syndrome coronavirus 2, transmembrane protease serine 2 (TMPRSS2), 3C-like protease (3CL^pro^/M^pro^), papain-like protease (PL^pro^), tamoxifen

## Abstract

Since 2019, severe acute respiratory syndrome coronavirus 2 (SARS-CoV-2) has been rapidly spreading worldwide, causing hundreds of millions of infections. Despite the development of vaccines, insufficient protection remains a concern. Therefore, the screening of drugs for the treatment of coronavirus disease 2019 (COVID-19) is reasonable and necessary. This study utilized bioinformatics for the selection of compounds approved by the U.S. Food and Drug Administration with therapeutic potential in this setting. In addition, the inhibitory effect of these compounds on the enzyme activity of transmembrane protease serine 2 (TMPRSS2), papain-like protease (PL^pro^), and 3C-like protease (3CL^pro^) was evaluated. Furthermore, the capability of compounds to attach to the spike-receptor-binding domain (RBD) was considered an important factor in the present assessment. Finally, the antiviral potency of compounds was validated using a plaque reduction assay. Our funnel strategy revealed that tamoxifen possesses an anti-SARS-CoV-2 property owing to its inhibitory performance in multiple assays. The proposed time-saving and feasible strategy may accelerate drug screening for COVID-19 and other diseases.

## 1. Introduction

At the end of 2019, a novel disease that causes severe acute respiratory syndrome (SARS), termed coronavirus disease 2019 (COVID-19), spread in Wuhan, China [1]. Because of its unexpected appearance and high transmissibility, this plague has caused a worldwide outbreak. Until 15 October 2021, there were 240 million confirmed cases and 4.9 million deaths reported worldwide (https://www.who.int/, accessed on 15 October 2021). SARS-coronavirus 2 (SARS-CoV-2) is the pathogen responsible for COVID-19. It belongs to the Orthocoronavirinae subfamily, commonly known as coronavirus, and is a positive single-stranded RNA virus with an envelope [1] that facilitates infection in mammals and birds.

The viral genome contains approximately 30,000 nucleotides encoding the spike protein (S), envelope protein (E), membrane protein (M), nucleocapsid protein (N), and structural proteins that maintain the structure of the virus. Infection and resistance to the host immune response are promoted by several proteins, such as ORF1ab and multiple accessory proteins [2]. The spike protein, a glycoprotein, is considered an important target for vaccine and drug design because of the infectious mechanism in which it is involved [3]. During the initiation of infection, the receptor-binding domain (RBD) of the spike protein binds to host membrane-associated angiotensin-converting enzyme 2 (ACE2), and transmembrane protease serine 2 (TMPRSS2) proteolytically cleaves and activates viral envelope glycoproteins [4]. The interaction of a spike with ACE2 promotes the fusion of the virus and the host cell membrane [5]. In addition to SARS-CoV-2, other types of coronaviruses and influenza viruses also rely on TMPRSS2 for entry into host cells [6,7].

Following entry, SARS-CoV-2 begins to replicate and proliferate. This process requires dozens of proteins and enzymes. Among them, the main protease (3C-like protease (3CL^pro^/M^pro^)) and papain-like protease (PL^pro^) are essential for virus replication [8]. 3CL^pro^ processes viral polyproteins [9] and plays a dominant role in viral replication [10]. Similarly, PL^pro^ is involved in the replication of a virus by facilitating the assembly of the replicase complex [11]. Therefore, TMPRSS2 [12], 3CL^pro^ [13], PL^pro^ [14], and RBD [15] have been recognized as targets for drug development.

Molecular docking is a computational method utilized to evaluate the binding possibility between ligand and target, such as ligand–protein docking [16]. The binding affinity usually represents the stability of the complex and is assessed by predicting intermolecular interaction [17]. Because of its advantages (i.e., low cost and high throughput), molecular docking has been applied to drug screening [18]. For instance, antagonists against SARS-CoV-2 have been screened using the AutoDock Vina program [19]. DockCoV2, an available database, records molecular docking information of SARS-CoV-2 target proteins and contains a compound library [20].

**Figure 1:**
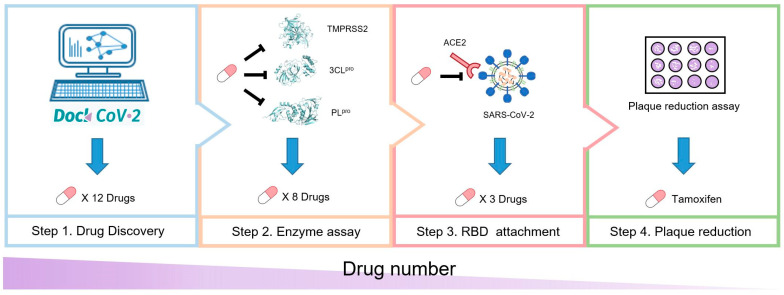
Study overview. A schematic of our anti-SARS-CoV-2 compound screening process. The anti-SARS-CoV-2 potential of 12 compounds was first analyzed through molecular docking using DockCoV2. Next, the inhibitory effect of those compounds on TMPRSS2, 3CL^pro^, and PL^pro^ was tested. The RBD attachment assay indicated the ability of the compound to block the interaction between the spike RBD and ACE2. Finally, the plaque reduction assay revealed the antiviral activity of the compounds.

According to their significance in the SARS-CoV-2 life cycle, we selected TMPRSS2, 3CL^pro^, PL^pro^, and RBD as target proteins for drug discovery. First, we utilized DockCoV2 as a molecular docking data resource and further analyzed the binding affinity and residue-scale binding pattern. We initially screened 12 drugs that have the potential to inhibit TMPRSS2, 3CL^pro^, or PL^pro^. Next, we tested the inhibitory effect of these 12 compounds on the enzyme activity of TMPRSS2, 3CL^pro^, and PL^pro^. We obtained 8 compounds with a half-maximal inhibitory concentration (IC_50_) of <50 μM for TMPRSS2, 3CL^pro^, or PL^pro^. Subsequently, we tested the ability of these 8 compounds to inhibit the binding between the spike protein and ACE2, obtaining 3 compounds with a half-maximal effective concentration (EC_50_) of <25 μM. Finally, we tested these 3 compounds using a plaque reduction assay, revealing that tamoxifen exerts a significant inhibitory effect on the infection and replication of SARS-CoV-2. The overall process is presented in Figure 1.

## 2. Materials and Methods

### 2.1. Materials

Vero E6 cells (CRL-1586) were obtained from the American Type Culture Collection (Manassas, VA, USA) and were grown in DMEM supplemented with 10% FBS and 1% antibiotic/antimycotic and incubated at 37 °C in a humidified atmosphere with 5% CO_2_. An SmBiT-ACE2-expressing cell line was established and cultured according to a previous publication [21]. FreeStyle™ 293-F cells, OPTI-MEM-1 buffer, Dulbecco’s Modified Eagle’s Medium (DMEM; 10569-044), fetal bovine serum (FBS; 10082-147), and antibiotic/antimycotic (15240-062) were purchased from Gibco (Medford, MA, USA). Afatinib (HY-10261), atorvastatin (HY-17379), homoharringtonine (HY-14944), mepacrine (HY-13735A), neratinib (HY-32721), rapamycin/sirolimus (HY-10219), tamoxifen (HY-13757A), and vemurafenib (HY-12057) were obtained from MedChem Express (Princeton, NJ, USA). Dactinomycin (101-50-76-0) was obtained from MDBio (Taipei, Taiwan). Doxorubicin (S1208) and niclosamide (S3030) were purchased from Selleckchem (Houston, TX, USA). Ethacrynic acid (SML1083), phenylalanyl chloromethyl ketone (PCK)-trypsin (T1426), and polyetherimide were obtained from Sigma-Aldrich (St. Louis, MO, USA). Furimazine was obtained from Aobious Inc. (Gloucester, MA, USA). The sequence of the SARS-CoV-2 spike gene (human codon optimized sequence and depletion of C-terminal 18 aa residues) was acquired from RNAi Core (Academia Sinica, Taipei, Taiwan). NanoLuc (including large binary technology (LgBiT) and small binary technology (SmBiT)) was acquired from Promega Corporation (Madison, WI, USA). The Twin-Strep-tag sequence was acquired from IBA Lifesciences GmbH (Göttingen, Germany). For the production of the Spike-RBD-LgBiT-TwinStrep fusion protein, spike protein signal peptide (MFVFLVLLPLVSSQ), codon-optimized spike-RBD sequence, (GGS)4-NanoLuc LgBiT sequence, and GA(ENLYFQG)SG-Twin-Strep-tag sequence were subcloned sequentially into a pAY5 expression vector (mAID-EGFP-NLS piggyBac) (a gift from Dr. Masato Kanemaki; Addgene #140532), and the mAID-EGFP-NLS sequence was removed. The B.1.617.1 RBD variant was produced by site-directed mutagenesis of L452R and E484Q. The results were obtained using a microplate reader (BioTek Synergy HTX, Santa Clara, CA, USA).

### 2.2. Methods

#### 2.2.1. Analysis and Visualization of Docking Results of 12 Candidate Compounds from DockCoV2

We evaluated 12 compounds approved by the U.S. Food and Drug Administration (FDA) from the DockCoV2 database (https://covirus.cc/drugs/, accessed on 26 June 2021) [20]. This database records docking results of drugs approved by the FDA and included in the Taiwan national health insurance scheme with several SARS-CoV-2-related proteins. We analyzed pose 1 (i.e., the strongest binding affinity pattern) of each compound with three target proteins (i.e., TMPRSS2, 3CL^pro^, and PL^pro^). PyMOL software was used to determine the binding patterns and compound–protein polar contact interaction [22].

#### 2.2.2. Expression and Purification of SARS-CoV-2 3CL^pro^

For the expression and purification of SARS-CoV-2 3CL^pro^, we followed our previously reported procedure [23]. The synthetic gene was cloned into pET32a vector encoding thioredoxin and the His-tag at the N-terminus of the target protein. The plasmid was transformed into *Escherichia coli* (*E. coli*) JM109 competent cells. Ampicillin-resistant colonies were selected and subsequently transformed into *E. coli* BL21(DE3) for protein expression. Overnight culture of a single transformant (5 mL) was added to 500 mL of fresh LB medium containing 100 μg/mL ampicillin. The cells were grown to an OD600 of 0.6 and added with 1 mM isopropyl-β-thiogalactopyranoside to induce recombinant protein production for 4–5 h. After induction, the cells were harvested by centrifugation at 7000× *g* for 15 min.

For purification of 3CL^pro^ conducted at 4 °C, the cell pellet was suspended in lysis buffer (40 mL) containing 25 mM Tris-HCl, pH 7.5, and 150 mM NaCl. To disrupt the cells, a French press was used. The cell-free extract was loaded onto a 10 mL Ni-NTA column equilibrated with the same buffer containing 5 mM imidazole. The column was washed with 5 mM imidazole and then by 30 mM imidazole-containing buffer. The His-tagged 3CL^pro^ was eluted with the lysis buffer with 300 mM imidazole. After overnight dialysis with the buffer, the tagged 3CL^pro^ was subjected to FXa protease treatment to remove thioredoxin and His-tag. The untagged protein mixture was loaded onto a Ni-NTA column and eluted with the buffer containing 5 mM imidazole. The eluted tag-free 3CL^pro^ was dialyzed and stored in the buffer of 12 mM Tris-HCl, pH 7.5, 120 mM NaCl, 0.1 mM ethylenediaminetetraacetic acid (EDTA), and 2 mM dithiothreitol (DTT) at −70 °C. For experiments, the protein concentrations were determined based on the 280 nm absorbance.

#### 2.2.3. Expression and Purification of SARS-CoV-2 PL^pro^

For the preparation of SARS-CoV-2 PL^pro^, our previously reported procedure was followed [23]. The synthetic gene of SARS-CoV-2 PL^pro^ was cloned into the pET16b vector, encoding His-tag at the N-terminus. The recombinant PL^pro^ plasmid was transformed into *E. coli* JM109 competent cells to select the 100 μg/mL ampicillin-resistant colony that was subsequently transformed into *E. coli* BL21(DE3) for protein expression. Overnight culture of a single transformant (5 mL) was added to 500 mL of fresh LB medium containing 100 μg/mL ampicillin. The cells were grown to an OD600 of 0.6 and added with 1 mM isopropyl-β-thiogalactopyranoside to induce protein production for 4–5 h. After induction, the cells were harvested by centrifugation at 7000× *g* for 15 min.

For purification of PL^pro^ conducted at 4 °C, the cell paste was suspended in lysis buffer (40 mL) containing 25 mM Tris-HCl at pH 7.5 and 150 mM NaCl. To disrupt the cells, a French press was used. The cell-free extract was loaded onto a 10 mL Ni-NTA column equilibrated with the lysis buffer containing 5 mM imidazole. The column was washed with 5 mM imidazole and then by 300 mM imidazole-containing buffer to elute the His-tagged PL^pro^. The His-tagged PL^pro^ was dialyzed using a buffer containing 12 mM Tris-HCl, pH 7.5, 120 mM NaCl, 0.1 mM EDTA, and 2 mM DTT and stored at −70 °C. For experiments, the protein concentrations were determined on the basis of 280 nm absorbance.

#### 2.2.4. Inhibition Assay of 3CL^pro^ and PL^pro^

The activity of 3CL^pro^ was monitored using a fluorogenic peptide, Dabcyl-KTSAVLQSGFRKME-Edans, as described in our previous report [23]. The fluorescence increase resulting from the substrate cleavage by 3CL^pro^ was followed with time at 538 nm upon excitation at 355 nm using a fluorescence plate reader. IC_50_ values of the active drugs were measured in reaction mixtures containing 35 nM 3CL^pro^ with a 6 μM fluorogenic substrate in a buffer of 20 mM Bis-Tris (pH 7.0) in the absence and presence of various concentrations of the inhibitors. Inhibition against PL^pro^ was measured using 75 nM enzyme with a 10 μM fluorogenic substrate, z-Arg-Leu-Arg-Gly-Gly-AMC, at an excitation of 355 nm upon emission of 460 nm in a buffer of 20 mM HEPES (pH 7.5) without and with various concentrations of the inhibitors as reported previously [23]. The initial velocities of the inhibited reactions were plotted against the different inhibitor concentrations to yield the IC_50_ value by fitting with the equation: A(I) = A(0) × {1 − [I/(I + IC_50_)]}, where A(I) is the enzyme activity with inhibitor concentration I, A(0) is the enzyme activity without an inhibitor, and I is the inhibitor concentration. For each data point, the measurements were repeated three times to yield the averaged number and the standard deviation.

#### 2.2.5. Expression, Purification, and Inhibition Assay of Human TMPRSS2

Cloning of TMPRSS2 was performed as previously reported [24]. The synthetic gene encoding the catalytic domain of human TMPRSS2 (residues 256–492) was cloned into the pMAL-c5X vector (New England Biolabs). This plasmid was transformed into *E. coli* BL21 (DE3) for protein overexpression. Overnight culture of a single transformant (5 mL) was added to 500 mL of fresh LB medium containing 100 mg/mL ampicillin. The cells were grown to an OD600 of 0.6 and added with 1 mM isopropyl-β-thiogalactopyranoside for further 20 h incubation at 16 °C for recombinant protein production. The cells were harvested by centrifugation at 7000× *g* for 15 min.

Purification of the maltose-binding protein (MBP)-tagged TMPRSS2 was conducted at 4 °C. The cell paste obtained from the cell culture (1 L) was suspended in a lysis buffer (40 mL) containing 25 mM Tris-HCl (pH 7.5) and 150 mM NaCl. To disrupt the cells, a French press was used. The cell-free extract was loaded onto a 10 mL amylose resin column (New England Biolabs, Ipswich, MA, USA) equilibrated with the lysis buffer. The column was washed with 10 column volumes of lysis buffer, and then the MBP-tagged TMPRSS2 bound to the amylose resin was eluted with the elution buffer (25 mM Tris, pH 7.5, 150 mM NaCl, and 10 mM maltose).

For assaying the inhibition of the purified MBP-tagged TMPRSS2 (residues 256-492) activity, a fluorogenic peptide substrate, Boc-Gln-Ala-Arg-AMC, was used with excitation at 355 nm and emission at 460 nm. The enzyme (0.22 μM) was preincubated in the presence or absence of various concentrations of inhibitors in 20 mM HEPES and pH 7.5 assay buffer for 10 min in black 96-well immuno plates (Thermo Scientific). Next, the fluorescent substrate (10 μM) was added and incubated for 60 min at room temperature. The reactions were monitored by using a fluorescence plate reader (Fluoroskan Ascent, Thermo Labsystems, Franklin, MA, USA). The initial velocities of the inhibited reactions were plotted against the different inhibitor concentrations to yield the IC_50_ value by fitting with the equation A(I) = A(0) × {1 − [I/(I + IC_50_)]}. In this equation, A(I) is the enzyme activity with inhibitor concentration I, and A(0) is the enzyme activity without an inhibitor. For each data point, the measurements were repeated thrice to yield the mean and standard deviation.

#### 2.2.6. RBD–ACE2 Attachment Assay

The RBD–ACE2 binding assay was established to monitor the interaction between the recombinant RBD protein and ACE2 with the application of NanoBiT technology [24]. We conducted the RBD–ACE2 attachment assay with slight modifications. Specifically, the recombinant RBD-LgBiT-TwinStrep fusion protein was produced in FreeStyle™ 293-F cells (Gibco™, Carlsbad, CA, USA) at 37 °C with 3 µg/mL polyetherimide (Sigma-Aldrich, St. Louis, MO, USA) induction for 7 days. After centrifugation, the supernatant was collected, and the purification of RBD-LgBiT-TwinStrep was conducted using the batch purification approach under native condition (IBA Lifesciences GmbH, Göttingen, Germany). To monitor the interaction between RBD and ACE2, SmBiT-ACE2-expressing cells [24] were seeded (1 × 10^4^ cells per well) and pretreated with indicated drugs (50 µL per well) for 5 min. Next, a reaction mixture (50 µL) containing 10 ng RBD-LgBiT-TwinStrep and 5 µM furimazine (Aobious Inc., Gloucester, MA, USA) in OPTI-MEM-1 buffer (Gibco™) was added to each well. The luminescence signal was recorded every 2 min and continuously for 45 min using a microplate reader (BioTek Synergy HTX, Santa Clara, CA, USA) at 37 °C and a time-lapsed kinetics program. For the calculation of RBD inhibition of all agents, luminescent data from the time point showing the highest signal in the negative control sample were selected. The following formula was used: inhibition (%) = [1 − (luminescence signal of test sample)/(luminescence signal of negative control sample)] × 100.

#### 2.2.7. Virus Preparation

The SARS-CoV-2 strain used was hCoV-19/Taiwan/NTU13/2020, whose original sequencing data were available on GISAID under accession ID EPI_ISL_413592. After amplification of the virus in the Vero E6 cells, the virus titer was determined by plaque assay for subsequent analysis.

#### 2.2.8. Plaque Reduction Assay

The plaque reduction assay was performed to determine the antiviral activity of the tested compounds against SARS-CoV-2 [25]. Briefly, Vero E6 cells were seeded in 24-well plates (2 × 10^5^ per well) 1 day before infection. Approximately 50–100 plaque-forming units (PFUs) of SARS-CoV-2 were added to the cell monolayer for 1 h at 37 °C. After removal of the viruses, the cell monolayer was washed once with PBS and overlaid with the media containing 1% methylcellulose with or without the test compound at indicated concentrations for 5 days. The cells were then fixed with 10% formaldehyde overnight, followed by staining of the cells with 0.5% crystal violet for the plaque counting. The percentage of inhibition was calculated using the formula [1 − (VD/VC)] × 100%, in which VD or VC refers to the virus titer in the presence or absence of the test compound at the indicated concentration, respectively. A dose-response curve was generated to calculate the IC_50_ using regression analysis of triplicated measurements.

## 3. Results

### 3.1. Selection of 12 Candidate Compounds and Identification of the Corresponding Interactions with TMPRSS2 Residues

**Figure 2:**
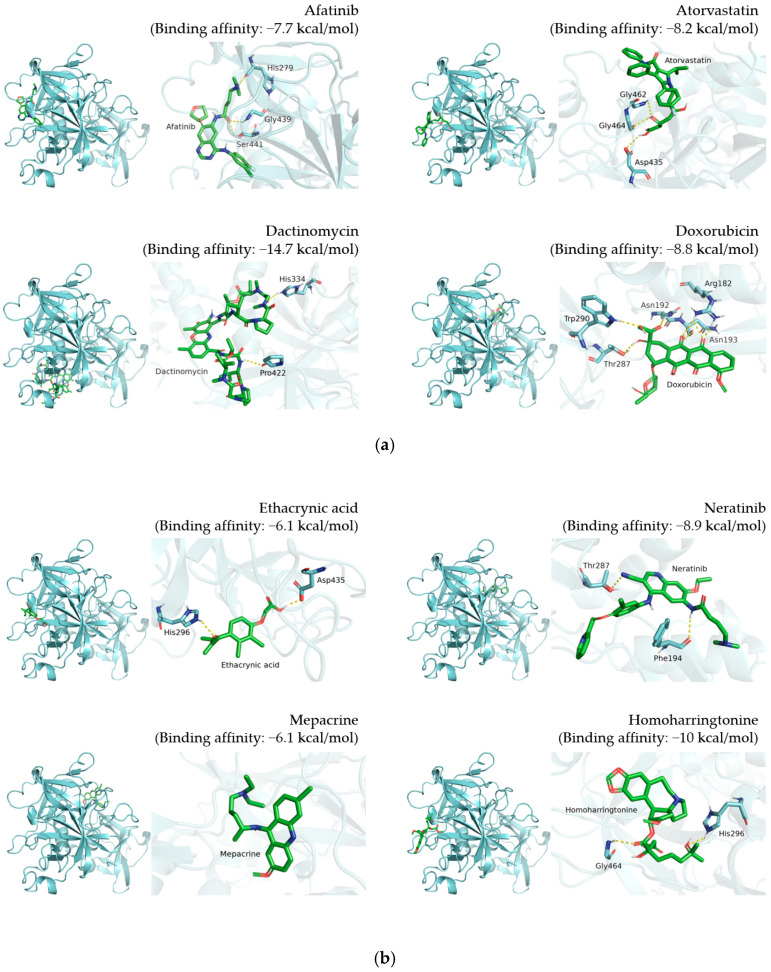

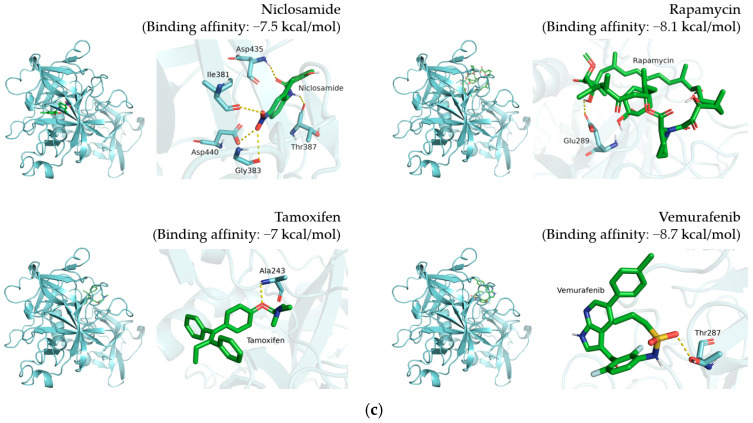
Docking results of 12 candidate compounds for TMPRSS2. (**a**) Afatinib, atorvastatin, dactinomycin and doxorubicin. (**b**) Ethacrynic acid, neratinib, mepacrine and homoharringtonine. (**c**) Niclosmide, rapamycin, tamoxifen and vemurafenib. These results present the pose 1 binding pattern of each compound in the whole protein and polar contact residue view. TMPRSS2 and compounds are shown in cyan and green, respectively. The polar contacts are marked with yellow dashes.

**Figure 3:**
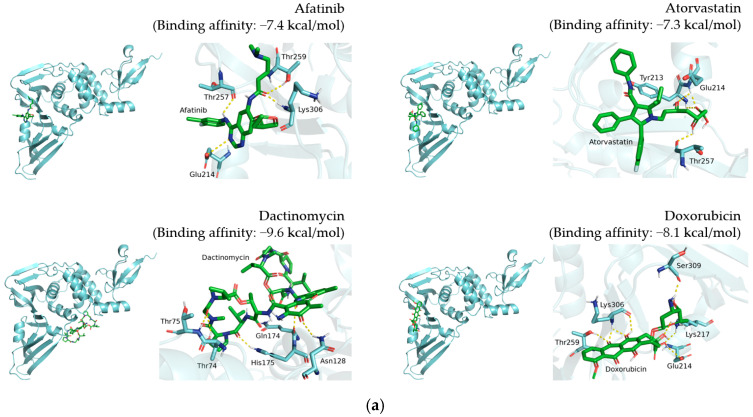

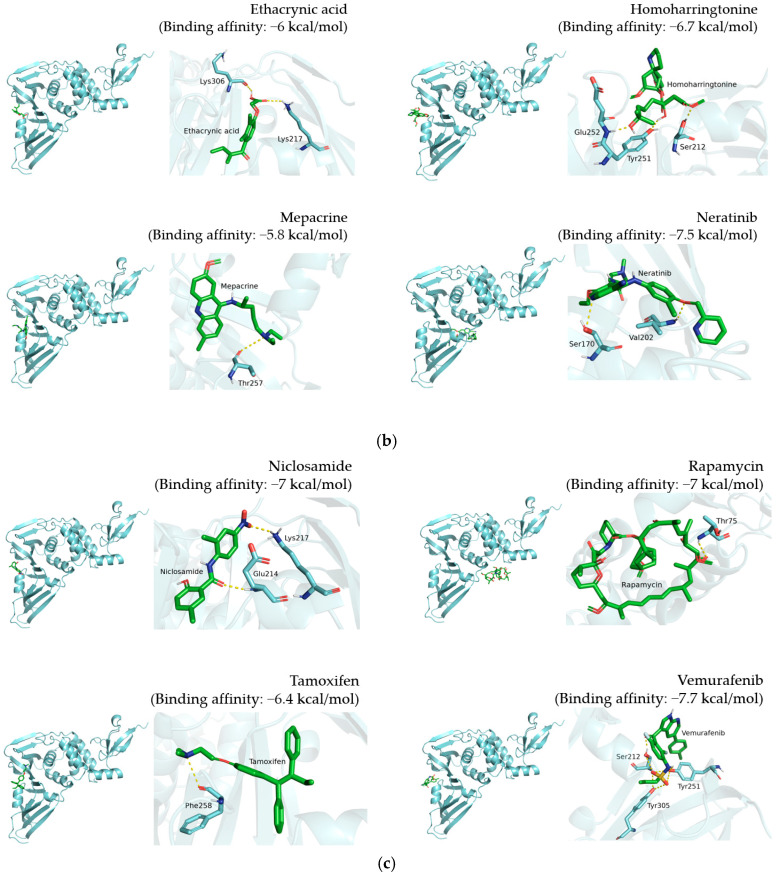
Docking results of 12 candidate compounds for PL^pro^. (**a**) Afatinib, atorvastatin, dactinomycin and doxorubicin. (**b**) Ethacrynic acid, homoharringtonine, mepacrine and neratinib. (**c**) Niclosamide, rapamycin, tamoxifen and vemurafenib. These results present the pose 1 binding pattern of each compound in the whole protein and polar contact residue view. PL^pro^ and compounds are shown in cyan and green, respectively. The polar contacts are marked with yellow dashes.

**Figure 4:**
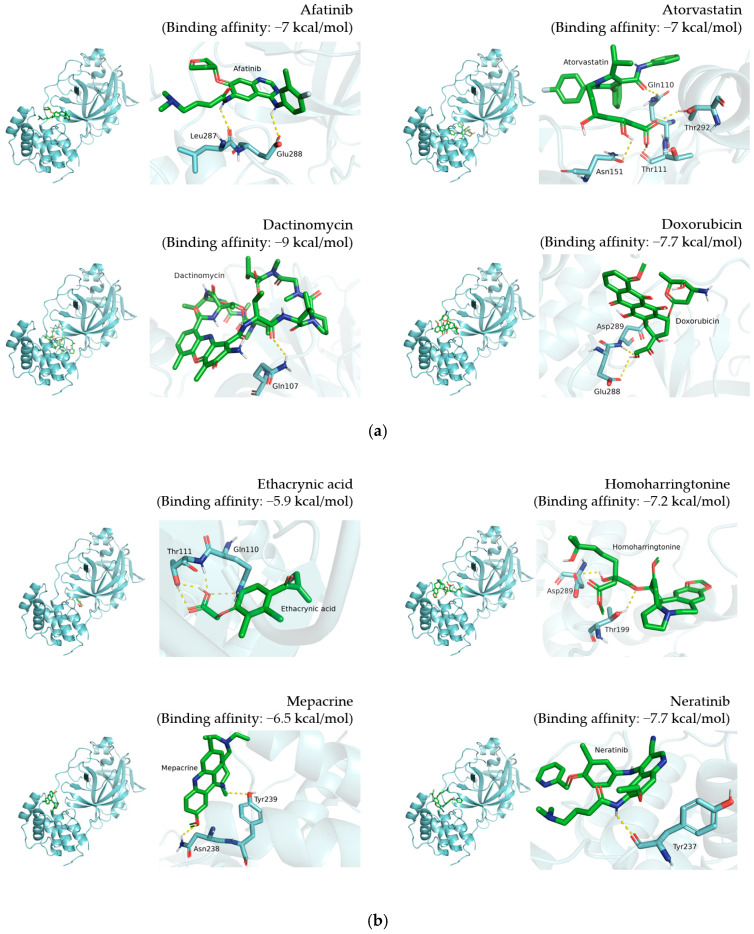

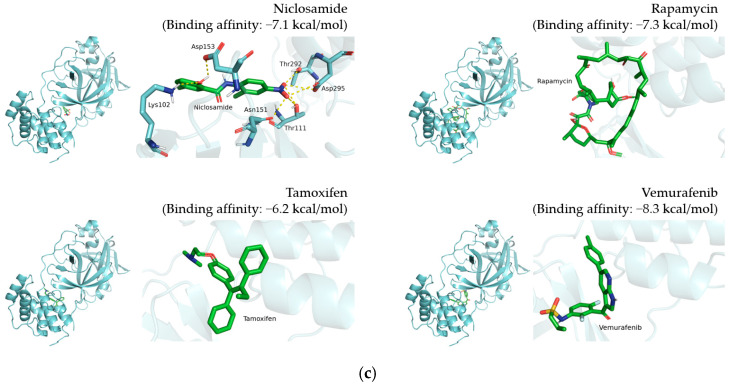
Docking results of 12 candidate compounds for 3CL^pro^. (**a**) Afatinib, atorvastatin, dactinomycin and doxorubicin. (**b**) Ethacrynic acid, homoharringtonine, mepacrine and neratinib. (**c**) Niclosamide, rapamycin, tamoxifen and vemurafenib. These results present the pose 1 binding pattern of each compound in the whole protein and polar contact residue view. 3CL^pro^ and compounds are shown in cyan and green, respectively. The polar contacts are marked with yellow dashes.

Visualization results display the whole protein–compound position and the polar contact residue schematic of TMPRSS2 (Figure 2), PL^pro^ (Figure 3), and 3CL^pro^ (Figure 4), respectively. The docking results are shown in Table 1, Table 2 and Table 3.

For TMPRSS2, the corresponding predicted binding affinities of doxorubicin, niclosamide, rapamycin, and tamoxifen were −8.8, −7.5, −8.1, and −7 kcal/mol, respectively (Figure 2 and Table 1). Furthermore, the residue-scale view reveals the potential amino acids involved in polar interaction. We observed that doxorubicin interacts with Arg182, Asn192, Asn193, Thr287, and Trp290 of TMPRSS2 with hydrogen bonds (Figure 2a), while niclosamide interacts with Ile381, Gly383, Thr387, Asp435, and Asp440 of TMPRSS2 through hydrogen bonding (Figure 2c). Rapamycin binds with Glu289 through hydrogen bonds, while tamoxifen interacts with Ala243 of TMPRSS2 (Figure 2c).

For PL^pro^, the predicted binding affinities of doxorubicin, niclosamide, rapamycin, and tamoxifen were −8.1, −7, −7, and −6.4 kcal/mol, respectively (Figure 3 and Table 2). Doxorubicin interacts with Glu214, Lys217, Thr259, Lys306, and Ser309 of PL^pro^ through hydrogen bonding (Figure 3a). Niclosamide binds to Glu214 and Lys217 of PL^pro^ (Figure 3c). Rapamycin and tamoxifen interact with Thr75 and Phe258 of PL^pro^, respectively (Figure 3c).

For 3CL^pro^, the binding affinities of doxorubicin, niclosamide, rapamycin, and tamoxifen were −7.7, −7.1, −7.3, and −6.2 kcal/mol, respectively (Figure 4 and Table 3). Doxorubicin interacts with Glu288 and Asp289 of 3CL^pro^. Niclosamide interacts with Lys102, Thr111, Asn151, Asp153, Thr292, and Asp295. However, there was no polar contact observed between 3CL^pro^ and rapamycin or tamoxifen. By visualizing the docking results, we dissected the potential binding residues involved in individual compound–protein hydrogen interaction. These interaction patterns may explain the antiviral validation results and provide evidence for determining important interaction positions.

### 3.2. Inhibition of TMPRSS2, 3CL^pro^, and PL^pro^ by 12 Candidate Compounds

By analyzing the molecular docking data, we assumed that these 12 compounds have the potential to attenuate the activity of TMPRSS2, PL^pro^, and 3CL^pro^. Therefore, we first performed the enzyme activity assay to evaluate the inhibitory effect of these compounds on individual target proteins. The threshold of IC_50_ was set at 50 μM. Compounds with an IC_50_ higher than the threshold were excluded from subsequent tests.

**Figure 5:**
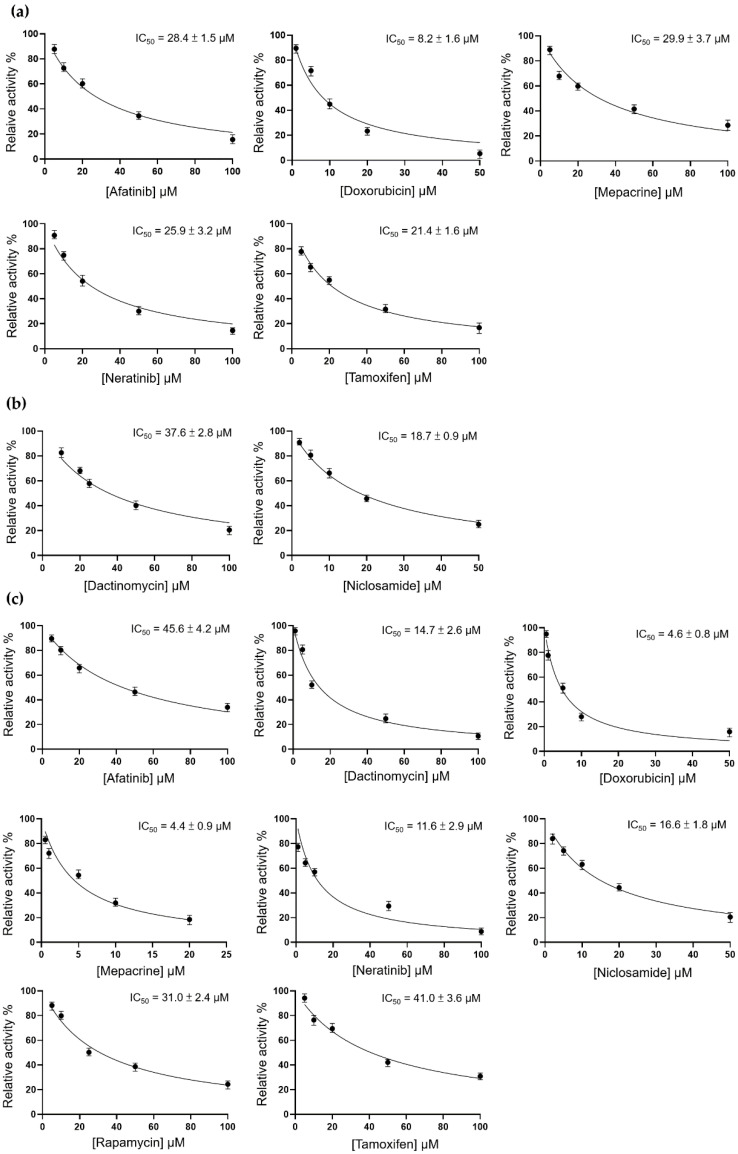
Inhibition assay for TMPRSS2, 3CL^pro^, and PL^pro^. (**a**) TMPRSS2 enzyme activity was measured using the following equation: A(I) = A(0) × {1 − [I/(I + IC_50_)]}. (**b**) 3CL^pro^ enzyme activity was calculated with the Michaelis–Menten equation. (**c**) PL^pro^ enzyme activity was calculated with the Michaelis–Menten equation.

For TMPRSS2, the IC_50_ values of afatinib, doxorubicin, mepacrine, neratinib, and tamoxifen were 28.4 ± 1.5, 8.2 ± 1.6, 29.9 ± 3.7, 25.9 ± 3.2, and 21.4 ± 1.6 μM, respectively (Figure 5a). For 3CL^pro^, dactinomycin and niclosamide were considered potential inhibitory compounds, with IC_50_ values of 37.6 ± 2.8 and 18.7 ± 0.9 μM, respectively (Figure 5b). For PL^pro^, we found that afatinib, dactinomycin, doxorubicin, mepacrine, neratinib, niclosamide, rapamycin, and tamoxifen exerted inhibitory effects with IC_50_ values of 45.6 ± 4.2, 14.7 ± 2.6, 4.6 ± 0.8, 4.4 ± 0.9, 11.6 ± 2.9, 16.6 ± 1.8, 31 ± 2.4, and 41 ± 3.6 μM, respectively (Figure 5c).

### 3.3. Inhibition of the Spike RBD for Wild-Type and B.1.617.1-Variant SARS-CoV-2 by Three Candidate Compounds

**Figure 6:**
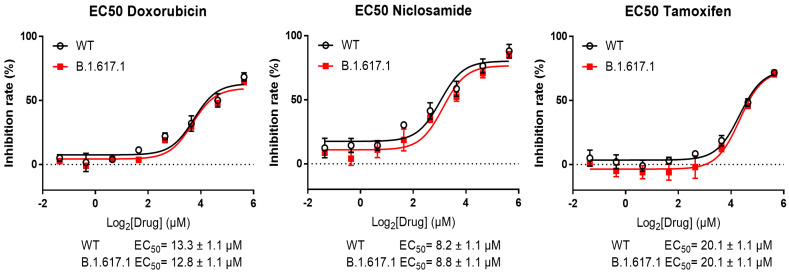
RBD attachment assay in wild-type and B.1.617.1 -variant SARS-CoV-2. EC_50_ profiles display the inhibitory effect of each compound on RBD attachment. The x-axis represents the compound concentration in log_2_ scale; the y-axis represents the inhibition rate.

Based on the previous enzyme activity assay, we tested the binding potency of afatinib, dactinomycin, doxorubicin, mepacrine, neratinib, niclosamide, rapamycin, and tamoxifen for the spike RBD. We expected that a lower EC50 would be associated with a higher possibility to block the interaction between the RBD and ACE2 and avoid the initiation of virus infection. The India variants are most transmissible SARS-CoV-2 lineages with mutations in the spike and other viral proteins. Therefore, we aimed to investigate and screen potential compounds for the treatment of cases infected with the India variant B.1.617.1. For this purpose, we performed RBD attachment assay for the above-mentioned compounds in both wild-type (WT) and B.1.617.1-variant SARS-CoV-2. In the WT test, tamoxifen, doxorubicin, and niclosamide exerted the top 3 inhibitory effects, with EC50 values of 20.1 ± 1.1, 13.3 ± 1.1, and 8.2 ± 1.1 μM, respectively (Figure 6). In the B.1.617.1-variant test, these values were 20.1 ± 1.1, 12.8 ± 1.1, and 8.8 ± 1.1 μM, respectively (Figure 6). These three compounds were further examined in the plaque formation assay to confirm their antiviral potency.

### 3.4. Verification with Plaque Reduction Assay for Three Candidate Compounds

Based on the results of the RBD analysis, we further used the plaque reduction assay to evaluate the antiviral activity of doxorubicin, niclosamide, and tamoxifen against SARS-CoV-2 in Vero E6 cells. Cells were pre-infected and treated with individual compounds for 120 h. After staining with crystal violet, the number of plaques was determined.

**Figure 7:**
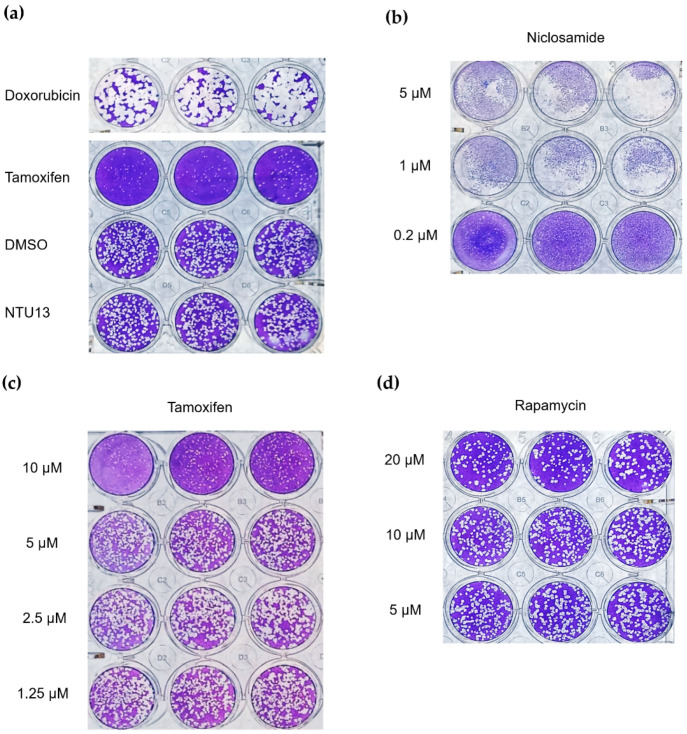
Plaque reduction assay for the candidate compounds. Infected cells were treated with individual compounds for 120 h to evaluate the antiviral activity. (**a**) Treatment with six compounds at a concentration of 10 μM. (**b**) Treatment with niclosamide at three concentrations. (**c**) Treatment with tamoxifen at four concentrations. (**d**) Treatment with rapamycin at three concentrations.

At a concentration of 10 μM, doxorubicin did not effectively inhibit the replication and infection of the virus compared with control. In contrast, tamoxifen robustly protected cells from the viral infection (Figure 7a), and this virus inhibition was dose dependent (Figure 7c). At a concentration of 0.2 μM, niclosamide demonstrated antiviral property; nevertheless, the cells appeared broken into tiny particles, revealing the cytotoxicity of niclosamide (Figure 7b). Even at a concentration of 20 μM, rapamycin did not exert a significant effect on the inhibition of viral infection (Figure 7d). Overall, the plaque reduction assay showed the high potential of tamoxifen at a concentration of 10 μM, with robust inhibition activity against SARS-CoV-2 and without apparent cytotoxicity.

## 4. Discussion

Bioinformatics has been widely utilized in drug discovery to evaluate the properties of drugs and drug–target relationships [26,27]. For instance, molecular docking often predicts the binding affinity and interaction patterns between molecules. This information assists researchers in realizing the binding position of compounds and screening the potential binding pocket of the target protein [28]. In this study, data obtained from the DockCoV2 database provided us with information regarding the affinity and binding position of each compound for TMPRSS2, PL^pro^, and 3CL^pro^. This information revealed that these 12 compounds have the potential to bind or even block the corresponding proteins. Inhibition of related targets can be employed as a treatment against SARS-CoV-2. Hence, we sought to examine the antiviral activity of these compounds. After a series of assays, we identified doxorubicin, niclosamide, and tamoxifen as potential therapeutic agents against SARS-CoV-2. Of those, tamoxifen offered the greatest promise for further in vivo testing and clinical research.

By analyzing predicted binding patterns, we can speculate the residues involved in compound–protein interaction and explore binding pockets to validate their relationship. To determine the possible binding pattern, we analyzed the docking results for four compounds (Figure 2, Figure 3 and Figure 4 and Table 1, Table 2 and Table 3). For TMPRSS2 docking, we found that doxorubicin interacts with Arg182, Asn192, Asn193, Thr287, and Trp290, while niclosamide interacts with Ile381, Gly383, Thr387, Asp435, and Asp440. Moreover, tamoxifen forms hydrogen bonds at Ala243, and rapamycin interacts with Glu289 through hydrogen bonding. Comparison showed similar binding patterns for doxorubicin, tamoxifen, and rapamycin; however, niclosamide exhibited a different pattern. For PL^pro^, doxorubicin interacts with Glu214, Lys217, Thr259, Lys306, and Ser309, while niclosamide binds to Glu214 and Lys217 of PL^pro^. Tamoxifen forms a hydrogen bond with Phe258, while rapamycin interacts with Thr75. Rapamycin displayed a binding pattern different from those of the other three compounds. For 3CL^pro^, doxorubicin interacts with Glu288 and Asp289, while niclosamide interacts with Lys102, Thr111, Asn151, Asp153, Thr292, and Asp295. However, tamoxifen and rapamycin did not generate hydrogen bonds with 3CL^pro^. The reason responsible for this observation may be that nonpolar interactions, such as hydrophobic interactions, also contribute to the scoring in molecular docking. A report showed that a patent SARS-CoV-1 3CL^pro^ inhibitor exerted its effect by occupying the hydrophobic interaction volume between 3CL^pro^ and other proteins [29]. We reasoned that tamoxifen and rapamycin may also mainly interact with 3CL^pro^ through hydrophobic interaction. Doxorubicin exhibited a distinct binding pattern compared with the other three compounds. The difference in binding patterns may be attributed to the molecular features of the molecule and protein [30].

The predicted residues in this investigation are different from those recorded in other studies [31,32,33]. Nevertheless, we assumed that compounds inhibit SARS-CoV-2 infection by binding to the former residues and triggering protein conformational change, which interferes with protein activity. Studies revealed that 3CL^pro^ [34] and PL^pro^ [35,36] present protein conformational changes that may be important for the catalytic mechanism. For TMPRSS2, molecular docking and molecular dynamics simulations have shown that conformational change occurs in the compound–protein complex [33]. Determining the structure of the compound–protein complex may be an option for the detailed investigation of the binding pattern.

During SARS-CoV-2 infection, the interaction between the spike protein (specifically RBD) and ACE2 triggers the fusion of the viral and host membranes [5]. According to the mechanism, designing blockades for the interaction of the spike with ACE2 becomes crucial for attenuating viral infection. For instance, this concept has been applied to the development of vaccines [37]. In the RBD attachment assay, tamoxifen, niclosamide, and doxorubicin demonstrated great capability for attachment to the spike RBD (Figure 6). These results indicate that these compounds have the potential to block the RBD and reduce the chance of RBD–ACE2 interaction, which impedes SARS-CoV-2 infection. In addition, a similar study showed that compound-induced inhibition of viral attachment may be associated with antiviral activity in SARS-CoV-2 infection [38].

According to the results of inhibition assay, eight compounds (i.e., afatinib, doxorubicin, mepacrine, neratinib, tamoxifen, dactinomycin, rapamycin, and niclosamide) can inhibit TMPRSS2, 3CL^pro^, or PL^pro^. Among them, mepacrine is an anthelmintic drug for malaria [39], rapamycin is an immunosuppressant [40], and the remaining compounds are chemotherapeutic agents [41]. In a report published by the American Health System Pharmacists Association, rapamycin and niclosamide are mentioned as drugs against COVID-19 (https://www.ashp.org/-/media/assets/pharmacy-practice/resource-centers/Coronavirus/docs/ASHP-COVID-19-Evidence-Table.ashx, accessed on 15 October 2021).

In our plaque reduction assay, we used Vero E6 cells as a virus cell infection model. Vero cell is a kidney epithelial cell lineage that was isolated from an African green monkey and plays a significant role in a virus infection model, while Calu-3 cell, a human lung epithelial cell line, acts as a lung-related infection model. When we survey on PubMed, the number of literature on Vero E6 cell associated with COVID-19, being 314, is higher than that on Calu-3 cell (human epithelial cells derived from lung) associated with COVID-19, being 133, indicating that Vero cell is more widely used as an infection model. According to previous studies, Vero cells have been applied in vaccine production [42] due to interferon-deficient-derived high capability for virus infection [43,44]. Furthermore, Vero cells were utilized for generating anti-SARS-CoV vaccine, which prompts high-titer neutralizing antibody in an immunized mice model [45]. Additionally, Vero cells were adopted for viral plaque reduction assay in SARS-CoV-2 research [46,47,48,49,50]. These reports indicate that Vero cells are suitable to validate our drug effect on the inhibition of virus infection. Although we have validated the inhibitory potency of our drugs using Vero cells, the test of the inhibitory impact in human epithelial cells should be further investigated.

Rapamycin (also termed sirolimus) is a macrolide that was isolated from *Streptomyces hygroscopicus* in Easter Island in 1972 [51]. In 1999, it was approved by the US FDA as an immunosuppressant for suppressing the immune response of patients who have undergone organ transplantation [52]. Rapamycin acts as a mechanistic target of rapamycin kinase (mTOR) inhibitor that regulates cell growth, proliferation, cellular movement, metabolism, and survival in mammals. In recent years, numerous studies revealed that sirolimus has therapeutic effects on many types of cancer [53]. In addition, it is established that mTOR is related to virus replication and protein synthesis [54]. In previous studies, rapamycin exerted a therapeutic effect on Middle East respiratory syndrome-related coronavirus (MERS-CoV) [55] and influenza A virus subtype H1N1 [56]. Other studies indicated that rapamycin interferes with virus replication and infection by acting on the host cell rather than the virus. Consequently, the treatment dosage is not influenced by the amount or mutation type of the virus [57]. In the present study, rapamycin inhibited PL^pro^ (Figure 5a), but it did not inhibit 3CL^pro^ or TMPRSS2. Furthermore, the results of the RBD attachment assay showed that rapamycin could not efficiently inhibit the interplay between the RBD and ACE2. Several reports have suggested that sirolimus is effective in the treatment of COVID-19; thus, we also investigated the virus plaque. The evidence indicated that rapamycin inhibits viral replication, but not viral infection, thereby explaining the lower effectiveness in inhibiting the virus versus tamoxifen (Figure 7c,d). In addition, previous studies have shown that the combination of rapamycin with other drugs results in greater effectiveness in the treatment of H1N1 [56]. Therefore, combination therapy may be more effective in the treatment of COVID-19 compared with monotherapy.

Niclosamide, a US FDA-approved anthelmintic, was discovered in 1958 [58]. Recently, niclosamide has been utilized as an antimetabolite, antibacterial agent, and anticancer agent in clinical practice. Research has indicated its capability to inhibit SARS-CoV-2 [59,60] through blockage of endocytosis [61] or inhibition of S-phase kinase-associated protein 2 (SKP2) to enhance autophagy [62]. Moreover, studies have reported an inhibitory property of niclosamide against 3CL^pro^ [63]. This study revealed that niclosamide inhibited the 3CL^pro^ and PL^pro^ activity in SARS-CoV-2 (Figure 5b). The RBD attachment assay revealed that niclosamide exerted an inhibitory effect on both WT and B.1.617.1--variant SARS-CoV-2 (Figure 6). However, we observed that the administration of niclosamide was associated with high cytotoxicity. This finding implies that drug concentration should be reconsidered during examination (Figure 7b). Similarly, other studies revealed that niclosamide can inhibit the virus at low concentrations [60]; this fact represents the limitation of niclosamide and potential dangers linked to its usage.

Doxorubicin was isolated from *Streptomyces peucetius* around Fort Monte, Italy, in the 1950s; it is currently used in the treatment of cancer [64]. The mechanism is initiated by the inhibition of topoisomerase II in DNA replication, thereby stopping the replication process [65]. Because of its capability to inhibit viral helicase and the spike protein, doxorubicin has been recognized as an antiviral medication [16,66]. In addition, as shown in the literature, SARS-CoV-2 is sensitive to methylglyoxal. Furthermore, doxorubicin inhibits SARS-CoV-2 by increasing glucose metabolism, which leads to methylglyoxal formation [67]. Our study demonstrated that doxorubicin inhibits TMPRSS2, PL^pro^ (Figure 5a,c), and the spike (Figure 6). However, the results of the plaque reduction assay did not show an inhibitory effect on SARS-CoV-2 (Figure 7a).

Tamoxifen is a nonsteroidal selective estrogen receptor modulator that was first discovered in 1962. It is structurally derived from diethylstilbestrol-like estrogens and antiestrogens [68]. At present, tamoxifen is included in the list of essential medicines of the World Health Organization [69]. This agent is mainly used to prevent and treat breast cancer [70]. In addition to its antiestrogen effects, it can enhance human natural killer (NK) activity in vitro [71]. Another study showed that it can also amplify cytotoxic T lymphocyte-, NK cell-, and lymphokine-activated killer-cell-mediated target cell lysis [72]. It can also inhibit parasites, fungi, bacteria, and other microorganisms [73]. In terms of antiviral activity, previous studies have shown that it is effective against the Ebola virus, human immunodeficiency virus (HIV), and hepatitis C virus. In terms of mechanism, tamoxifen directly blocks estrogen receptors, causes growth source deficiency, and kills breast cancer cells. By influencing C-reactive protein, tamoxifen kills cancer cells or inhibits their growth [74] or increases the levels of nuclear factor erythroid 2-related factor 2 (Nrf2) to cause cell oxidative stress [75]. In addition, previous studies have pointed out that the androgen receptor (AR) signal controls the expression of TMPRSS2 [76], and TMPRSS2 is necessary for the SARS-CoV-2 spike protein to infect the human body, so androgen receptor inhibitors are used and have therapeutic potential for COVID-19. Interestingly, tamoxifen can bind to AR and inhibit its activity [77,78]. In addition to androgens, estrogen also regulates the expression of TMPRSS2 [79], so the use of tamoxifen may suppress the expression of TMPRSS2 and further reduce the infectivity of SARS-CoV-2. Other studies have revealed that tamoxifen inhibits mitochondrial complex 1 to inhibit cell growth [80] or adjusts the inhibitor tumor cell growth factor to inhibit tumor growth [81]. In terms of antiviral activity, tamoxifen inhibits the replication of HIV by activating protein kinase C (PKC) [82] and inhibits that of hepatitis C virus by affecting the estrogen receptor [83].

The in vitro effect of tamoxifen on anti-SARS-CoV-2 is evaluated in our study. This antiviral response in an animal model still requires further investigation. The interesting impact between in vitro and in vivo can be distinct due to the difference in biological complexity, such as metabolic capability [84]. Although a cancer study revealed that tamoxifen enhances apoptosis with similar effectiveness in both in vitro and in vivo during gemcitabine combination [85], another study showed that the appropriate tamoxifen treatment time for the maximal inhibition of hepatocyte proliferation is different in in vitro and in vivo observation [86]. These studies imply that in vitro and in vivo validation may have a different impact. In this study, we investigate the inhibitory impact of tamoxifen on SARS-CoV-2 with an in vitro system. The detailed drug response, metabolism, duration, and comprehensive influence will be examined in vivo. The present results show that tamoxifen inhibits PL^pro^ and TMPRSS2 (Figure 5a,c) and effectively blocks the spike RBD (Figure 6). In the virus plaque experiment, we observed that tamoxifen significantly inhibited SARS-CoV-2 infection (Figure 7c). Although doxorubicin inhibited the 3CL^pro^ and TMPRSS2 enzyme activity and blocked the RBD, it failed to effectively inhibit SARS-CoV-2 compared with tamoxifen in the plaque reduction assay (Figure 7a). This observation may be attributed to the variety of antiviral mechanisms associated with tamoxifen and its half-life of 5–7 days [87]; of note, the half-life of doxorubicin is 29–35 h [88].

## 5. Conclusions

We used data from the DockCoV2 database to predict potential drugs with multiple targets and further tested the inhibitory effect on SARS-CoV-2. Finally, the virus plaque test validated tamoxifen as the drug with the greatest therapeutic potential. The selective screening strategy is a highly effective and practical strategy because it assists us in identifying drugs with therapeutic potential during an epidemic. Its advantages include rapid processing and limited requirement of resources for the development of new treatments. The compounds we screened in this study have FDA certification and have multiple disease targets. These features improve the safety of medications and reduce the risk of drug–drug interaction caused by combination treatment. In the future, research on the selection of disease targets and elucidation of drug mechanisms may enhance our understanding of conditions, thereby rendering the screening strategy more rapid and accurate.

## Figures and Tables

**Table 1:** Docking affinity for TMPRSS2, molecular structure of individual compounds, and TMPRSS2 residues involved in hydrogen interaction.

Compound Name	Compound Structure	TMPRSS2 Residues Involved in Hydrogen Interaction
Afatinib	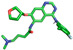	His279, Gly439, Ser441
Atorvastatin	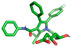	Asp435, Gly462, Gly464
Dactinomycin	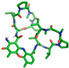	His334, Pro422
Doxorubicin	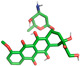	Arg182, Asn192, Asn193, Thr287, Trp290
Ethacrynic acid	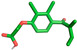	His296, Asp435
Homoharringtonine	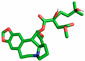	His296, Gly464
Mepacrine	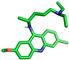	None
Neratinib	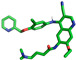	Phe194, Thr287
Niclosamide	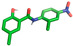	Ile381, Gly383, Thr387, Asp435, Asp440
Rapamycin	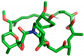	Glu289
Tamoxifen	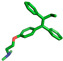	Ala243
Vemurafenib	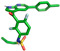	Thr287

**Table 2:** Docking affinity for PL^pro^ and molecular structure of individual compounds.

Compound Name	Compound Structure	PL^pro^ Residues Involved in Hydrogen Interaction
Afatinib	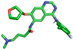	Glu214, Thr257, Thr259, Lys306
Atorvastatin	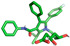	Tyr213, Glu214, Thr257
Dactinomycin	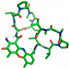	Thr74, Thr75, Asn128, Gln174, His175
Doxorubicin	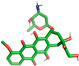	Glu214, Lys217, Thr259, Lys306, Ser309
Ethacrynic acid	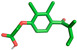	Lys217, Lys306
Homoharringtonine	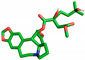	Ser212, Tyr251, Glu252
Mepacrine	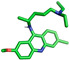	Thr257
Neratinib	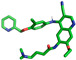	Ser170, Val202
Niclosamide	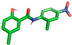	Glu214, Lys217
Rapamycin	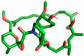	Thr75
Tamoxifen	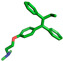	Phe258
Vemurafenib	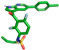	Ser212, Tyr251, Tyr305

**Table 3:** Docking affinity for 3CL^pro^ and molecular structure of individual compounds.

Compound Name	Compound Structure	3CL^pro^ Residues Involved in Hydrogen Interaction
Afatinib	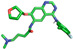	Leu287, Glu288
Atorvastatin	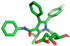	Gln110, Thr111, Asn151, Thr292
Dactinomycin	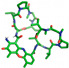	Gln107
Doxorubicin	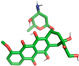	Glu288, Asp289
Ethacrynic acid	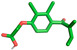	Gln110, Thr111
Homoharringtonine	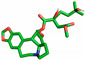	Thr199, Asp289
Mepacrine	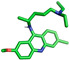	Asn238, Tyr239
Neratinib	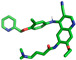	Tyr237
Niclosamide	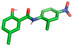	Lys102, Thr111, Asn151, Asp153, Thr292, Asp295
Rapamycin	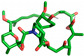	None
Tamoxifen	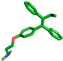	None
Vemurafenib	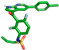	None

## Data Availability

The data presented in this study are available upon request from the corresponding authors.
